# The efficacy of community‐led monitoring: successes, lessons learnt and opportunities for improvement from the Zimbabwean context

**DOI:** 10.1002/jia2.70053

**Published:** 2025-10-11

**Authors:** Morgen Chinoona, Jephias Matunhu, Donald Denis Tobaiwa, Kudzaishe Mutungamiri, Melody Musendo, Tinashe Marange, Tinashe Chidede

**Affiliations:** ^1^ FACT Zimbabwe Harare Zimbabwe; ^2^ TMMRI Midlands State University Zvishavane Zimbabwe; ^3^ Jointed Hands Welfare Organization Gweru Zimbabwe

Community‐led monitoring (CLM) is vital in the global HIV response as it enables community participation and evidence‐based advocacy for improved health service delivery. CLM is “an accountability mechanism for HIV responses at different levels, led and implemented by community‐led organizations of people living with HIV (PLHIV), networks of key populations (KP), other affected groups” [1]. It is a form of social accountability, where citizens hold duty bearers accountable for the services they provide. Evidence highlights its effectiveness in bridging gaps between healthcare providers and communities, addressing systemic inequities and strengthening accountability [2]. The Global Fund to Fight AIDS, Tuberculosis and Malaria (GF) supported CLM coordinated by Family AIDS Caring Trust (FACT) commenced in 2021, driving community‐led action to improve services at 246 health facilities in 21 districts of Zimbabwe. It was initiated by civil society organizations in collaboration with PLHIV and KP communities, who determined its scope and priorities. Districts were consultatively selected with consideration of epidemiological burden and CLM coverage. CLM targets PLHIV, adolescent girls and young women and KP, including sex workers, men who have sex with men and sexual minorities. It involves 718 community health monitors (CHMs) selected by communities based on representation and levels of their literacy and commitment. CHMs utilize Kobo Collect surveys and community score cards to monitor availability, accessibility, acceptability, appropriateness and quality of HIV‐health services. Data are collected and synchronized from various tools, drawing insights that are disseminated and actioned on a quarterly basis at the facility and district levels.

CLM recognizes that, while HIV prevalence declined from 12.6% in 2019 to 10.5% in 2023 in Zimbabwe, some subpopulations remain behind [3]. Stigma and discrimination remain high, with the 2022 PLHIV Stigma Index noting 77.7% of sex workers reporting HIV status‐related stigma and 17.9% PLHIV discontinuing Antiretroviral Therapy (ART) in the preceding year due to stigma [4]. Stigma and legal barriers disproportionately hinder KP's access to equitable HIV services owing to Zimbabwe's socio‐cultural landscape dominated by Christian (85.3%) and traditional beliefs [3].

In this context, this article aims to (1) highlight CLM's achievements in improving access and uptake of HIV and sexual and reproductive health services, and (2) share experiences from CLM implementation in Zimbabwe, highlighting lessons and opportunities for improvement.

As highlighted in Table 1, between 2023 and 2025, CLM improved access to HIV/AIDS services, enhanced healthcare staff attitudes and strengthened linkages between healthcare facilities and communities. CLM has been instrumental in resolving stock‐outs and promoting differentiated service delivery. Nevertheless, its efficacy varies across districts, depending on the responsiveness of local health authorities. Regrettably, the gains are under threat as recent global health funding cuts beget the emergence of long queues and waiting times at health facilities for HIV services due to panic and cessation of community ART refill since March 2025. An increased shortage of antibiotics has also been reported across districts.

**Table 1:** Achievements of CLM

Achievement	Key findings and challenges	Actions taken and impact
Improved access to HIV/AIDS services	‐ 45% of respondents reported low service access due to long distances (>10 km) to health facilities.	‐ CLM led to the restoration of health posts in Nyanga and Chimanimani districts, thus reducing distance decay and improving access.
Treatment support and adherence	‐ 80+ interrupters recorded in 2023. ‐ Farmworkers risked losing food hampers if they miss work to collect ART.	‐ Reconnected 70 clients to ART (87% re‐initiation rate). ‐ Establishment of more ART refill groups now 200+ farm workers in Marondera access ART through these groups.
Improved staff attitudes	‐ Negative perceptions of nurses towards KPs. ‐ Understaffing led to long waiting times (>1 hour), contributing to service dissatisfaction.	‐ Communities self‐reported improved nurses’ attitudes towards KPs due to continuous engagement. ‐ Communities advocated for more nurses. While additional primary care nurses were recruited in some facilities, attribution could not be ascertained.
Resolving stock‐outs	‐ 30% (1109/3658) of respondents reported unavailability of essential medicines in Q4 2023. ‐ Shamva community resorted to unregistered vendors for medicines.	‐ Reported illegal medicine vendors. ‐ Improved medicine availability in clinics.
Addressing HIV testing kits shortages	‐ Stock‐outs and expired commodities hinder HIV Testing Services (HTS) in rural areas. ‐ 12% of respondents in Kariba district reported testing kit stock‐outs in Q4 2023.	‐ Empowered communities to report stock‐outs to district authorities. ‐ Improved response turnaround, from a month to about a week's time.
Differentiated service delivery for AGYW	‐ Limited trained nurses for long‐acting reversible contraceptives ‐ Youth reluctance to access SRH services ‐ Teenage pregnancies and early marriages prevalent (22% national rate)	‐ CLM advocated and facilitated youth‐friendly services (dedicated rooms and days for family planning service for AGYW).

Abbreviations: AGYW, adolescent girls and young women; AIDS, acquired immunodeficiency syndrome; ART, antiretroviral therapy; CLM, community‐led monitoring; HIV, human immunodeficiency virus; KPs, key populations; SRH, sexual and reproductive health.

***Source***: Authors’ analysis of CLM data 2023–2025.

However, sustainability of CLM in Zimbabwe is compromised by external funding dependence, hence its viability beyond donor funding is uncertain. Therefore, innovative local financing, reporting and engagement mechanisms are needed for sustainability. Whilst, this paper did not assess the return on investment (ROI) for CLM, to advance the CLM investment case, ROI analysis is essential to highlight its health and social benefits. Similarly, there is notable progress, where duty bearers are responsive, yet capacity building of communities remains crucial to improve data quality, utilization and advocacy. Furthermore, systematic engagements with policymakers are critical to ensure that CLM findings inform policies and programming, ultimately enhancing their impact and sustainability.

**Lessons include**:

- Zimbabwe‐CLM sustainability requires strong local funding mechanisms, engagement structures and reporting systems, as evidenced by recent global funding cuts.
- Effective advocacy requires additional resources and enhanced community capacities to drive lasting and sustainable CLM impact.
- While treatment and care are critical in ending AIDS, equal attention is needed on prevention services.
- CLM thrives within inclusive health systems, promoting acceptance and reducing discrimination across all sub‐groups.

**Opportunities for improvement**

- Expanding CLM beyond HIV strengthens social accountability in the health delivery system, fostering transparency and responsiveness in healthcare services in Zimbabwe.
- Building on the successes in the 21 districts supported by GF, there is a clear opportunity to scale up CLM to more health facilities in Zimbabwe.
- Investing in capacity building of communities on social accountability and advocacy empowers individuals and organizations to effectively influence health policies and decisions, fostering strengthened community systems for health.

CLM proved effective in enhancing access to HIV/AIDS services and in addressing systemic healthcare challenges in Zimbabwe. District‐level successes, such as better treatment adherence, resolved stock‐outs and stronger community engagement, demonstrate CLM's efficacy in fostering accountability and bridging gaps between communities and health service providers. However, its sustainability in Zimbabwe is threatened by reliance on external funding, including recent cuts from PEPFAR and the downsizing of GF support. To ensure long‐term impact, Zimbabwe must invest in local funding mechanisms, engage policymakers and build community capacities. Thus, without strategic investment, CLM's progress and potential could be significantly undermined. Expanding CLM beyond HIV and with wider geographical coverage, while promoting inclusivity, will also enhance its relevance, effectiveness and public acceptance. Furthermore, advocacy and active community participation remain critical to sustain health improvements, safeguard the gains and protect CLM from reprioritization risks.

## COMPETING INTERESTS

The project was funded by the International AIDS Society (IAS). The authors otherwise have no competing interests to declare.

## AUTHOR CONTRIBUTIONS

All the contributors were involved in extracting data from the reports and interpreting it for this study. The team was led by MC.

## FUNDING

The CLM program is funded by the Global Fund to Fight, AIDS, TB and Malaria, Data Analysis and publication was funded by the International AIDS Society with grant from Gates Foundation ‐ grant number INV‐049564.

## Data Availability

Data sharing is not applicable to this article as no datasets were generated. However, the CLM programme data is available at FACT Zimbabwe for reference.
